# College Campus Food Pantry Program Evaluation: What Barriers Do Students Face to Access On-Campus Food Pantries?

**DOI:** 10.3390/nu14142807

**Published:** 2022-07-08

**Authors:** Francilia de K. Brito-Silva, Wanyi Wang, Carolyn E. Moore, Cynthia Warren, Derek C. Miketinas, Wesley J. Tucker, Kathleen E. Davis

**Affiliations:** 1 Department of Nutrition and Food Sciences, Texas Woman’s University, Science and Research Complex 204, P.O. Box 425888, Denton, TX 76024, USA; cwarren2@twu.edu (C.W.); kdavis10@twu.edu (K.E.D.); 2 Center for Research Design and Analysis, Texas Woman’s University, 6700 Fannin, Houston, TX 77030, USA; wwang@twu.edu; 3 Department of Nutrition and Food Sciences, Texas Woman’s University, 6700 Fannin, Houston, TX 77030, USA; cmoore8@twu.edu (C.E.M.); dmiketinas@twu.edu (D.C.M.); wtucker1@twu.edu (W.J.T.)

**Keywords:** food security, food insecurity, college students, food pantry, on-campus food pantries, hungry, barriers, food pantry barriers

## Abstract

The purpose of this study was to explore barriers Texas Woman’s University (TWU) students face in accessing on-campus food pantries. This cross-sectional, survey-based study was conducted in Fall 2021. Students’ use of the food pantries and barriers to utilization, including qualitative questions, were evaluated using descriptive statistics and thematic analyses. Students (*n* = 529) completed the survey. Despite a high prevalence of food insecurity (49.2%), most students reported never using the pantries (89.8%). Almost half of the students were unaware that these pantries existed on campus (47.8%). More than one in four students believed there were barriers to accessing the pantries, with time tissues, lack of transportation, limited food pantry hours of operation, and social stigma most commonly cited as major barriers to access. Food insecurity remains an urgent problem at TWU since the prevalence has remained high since 2019 despite the institutions’ efforts to reduce it. One of those resources has not been utilized as expected, which might impede improvements in food security among students. TWU on-campus food pantries can use these findings to address major barriers by offering after-hours access through the libraries or campus police, partnering with public transportation, and normalizing accessing food assistance.

## 1. Introduction

Food insecurity is a major burden among college students [1,2]. The prevalence of food insecurity in college students ranges from 10% to 75%, with an average estimate of 41% across the United States [2]. Because food insecurity is associated with many life stressors such as poor mental [3,4] and physical health [1], reduced academic performance [4,5,6], and overweight and obesity [7], the literature indicates that interventions to mitigate food insecurity are warranted [8]. One widely used strategy is to provide free food resources to people with food insecurity to alleviate immediate needs [9,10,11]. Generally, this type of assistance is delivered via food pantries [12]. However, few college students with food insecurity take advantage of on-campus food pantries [11,13].

According to the U.S. Code, a food pantry means “a public or private nonprofit organization that distributes food to low-income and unemployed households, including food from sources other than the Department of Agriculture, to relieve situations of emergency and distress” [14]. Normally food pantries are sourced by food banks and other nonprofit organizations that serve as food distribution warehouses. For example, the Texas Woman’s University (TWU)’s food pantries work in partnership with local food banks and accept food donations as well, offering mostly non-perishable food items [15,16]. Although shelf-stable food is more likely to be offered by food pantries, they are encouraged to provide fresh produce and to ensure whole grains, dairy, spices, and condiments are available [17]. Studies to date have shown college food pantries to be consistently underutilized [11,13]. Low utilization has been attributed to barriers such as social stigma, inconvenient hours of operation, self-identity [11], food pantry location [13], and lack information on how to access the pantries [11,13].

Food insecurity among college students may differ throughout the United States [2], as well as food pantry use and barriers to use, with differences occurring according to the characteristics of the student body. Thus, it is important to determine the barriers to using on-campus food pantries faced by each institution’s student body as a first step to serving the student community better and thus mitigating food insecurity on campus. Therefore, the purpose of this study was to explore the barriers TWU Denton students faced when using the TWU on-campus food pantries. This study had five aims: (1) to determine the current prevalence of food insecurity among TWU Denton students and describe demographic characteristics; (2) to determine what demographic factors are associated with the prevalence of food insecurity among students; (3) to describe the students’ profile of on-campus food pantries awareness, use, satisfaction, and barriers; (4) examine students’ awareness and use by students’ food security status and demographic factors; and (5) assess the availability of healthy food in one of the on-campus food pantries by conducting an inventory.

## 2. Materials and Methods

### 2.1. Design

This cross-sectional, online survey-based study was conducted during Fall 2021 at the Texas Woman’s University (TWU) Denton campus. The survey assessed TWU students’ use of on-campus pantries and barriers to utilization, including qualitative questions. It was approved by the TWU Institutional Review Board (IRB). The study survey covered the following sections: screening questions (being 18 years old and older and being a TWU Denton student); demographic characteristics (gender, age, race/ethnicity, enrollment status, and living arrangements); food security status; and food pantry awareness, utilization, and barriers to use. In addition, an inventory of the school-sponsored food pantry was completed by the research team based on the Cooking Matters in your Food Pantry Toolkit [18].

### 2.2. Participants and Recruitment

The research team sent an initial email invitation to all TWU Denton campus students included in the TWU listserv. Three weekly follow-up email reminders were sent thereafter. Students who were ≥18 years old and were currently enrolled as a student at the TWU Denton campus were considered eligible to participate. All students who completed the survey were invited to enter their email addresses to have a chance to win one of 30 drawings for a USD 50 gift card.

### 2.3. Survey

This study employed an internet-based survey delivered using PsychData (PsychData LLC, State College, PA, USA) composed of a mix of questions from validated surveys. First, food security status was assessed using the USDA Food Security Module Six-item Short Form. Nineteen questions about food pantry awareness, use, and barriers to access on-campus food pantries were based on a validated survey with slight modifications to serve the TWU student body [11]. The food pantry survey asked students whether they were aware TWU had food pantries, whether they have used the pantries, whether they were satisfied with the food pantries’ items, what food items they would like the food pantries to offer, and what suggestions they have to improve the food pantries, and finally whether they thought there were barriers to access the food pantries. Students who reported barriers to access the pantries were asked to name those barriers. This study also involved an inventory of food items available in one of the pantries based on the Cooking Matters in your Food Pantry Toolkit [17], which presents a list of healthy food items food pantries are recommended to have available. The inventory evaluated the availability of these healthy foods at baseline, mid-semester, and end-of-semester and was completed by the researcher.

### 2.4. Data Analysis

The data were examined for invalid and implausible data to identify and correct any errors. Next, the percentages and pattern of the missing data on all closed-ended questions, except questions involving logic, were examined. There were 0.4% missing data, and it was missing completely at random. Normality was assessed using histograms, and outliers were assessed using boxplots to see if parametric analysis assumptions were met. In order to determine the current prevalence of food insecurity among TWU Denton students and their demographic characteristics, descriptive statistics were used. Specifically, food security status was classified as high, low, or very low food security based on the scoring guide of the USDA food security module six-item short form [19]. In order to determine what demographic factors (age, race, school year, living arrangement, work hours per week, and having children under the age of 18 in the household) affected the prevalence of food insecurity, a multinomial regression model was performed. In order to describe the students’ on-campus food pantries awareness, use, satisfaction, and barriers to access, descriptive statistics and qualitative thematic analysis were conducted. Finally, to examine which demographic factors and food security status predict the use and awareness of on-campus food pantries, binary logistic regression models were performed. The quantitative analysis was conducted using SPSS v28 (Armonk, NY, USA: IBM Corp.), and the thematic analysis using NVivo 12 (QSR International, Burlington, MA, USA).

## 3. Results

Out of 13,666 students on the TWU Denton listserv, including all-year classifications, 575 students answered the survey (response rate of 4.2%). After excluding students who did not meet the inclusion criteria, duplicated cases, and invalid cases, a final sample of 529 students was included in the study. The student sample was mainly female (93.9%). Most of the participants were under 25 years old (69.1%) and highly diverse, with non-White students representing 59.9%. Additionally, the majority of the respondents were considered full-time students (84.7%) and living off-campus (72.4%; Table 1).

### 3.1. Food Security Status and Predictors

Half of the participants were high food secure (50.9%) while the other half were food insecure (49.2%), with food-insecure students divided evenly between low food security and very low food security (24.6% in each sub-group; Table 1). Multinomial logistic regression was conducted to predict food security status from the following demographic factors: age, race, school year, living arrangement, work hours per week, and having children under the age of 18 in the household. High food security was used as a reference to be compared with low food security and very low food security. The results showed that the overall model was significant (χ^2^(28) = 50.06, *p* = 0.005, Nagelkerke R^2^ = 0.112; Table 2). When comparing low food security with high food security, none of the independent variables predicted low food security status. However, very low food security was predicted by race and education. Specifically, students who identified as multiple races were 2.22 times more likely than White students to be very low food secure than high food secure status (OR = 2.22, *p* = 0.030). Undergraduate students were more likely to be very low food secure than graduate students (sophomore OR = 4.96, *p* = 0.013, junior OR = 5.21, *p* = 0.006, and senior OR = 5.01, *p* = 0.004) as compared to high food secure (Table 2).

### 3.2. Food Pantry Use, Awareness, and Satisfaction

The descriptive statistics showed that almost half of the students were not aware of the on-campus food pantries’ services (47.8%), and the majority of the students had never used the pantries (89.8%). Of the small number of students who had used the pantries, the majority (93.8%) were satisfied with either or both of the food pantries’ services (Table 3). Due to the very small sample size in each level of students who used the food pantry, they were combined into one category as “Yes” to be compared with students who did not use the food pantry.

Logistic regression analysis performed to assess the effects of demographic factors and food security status on the likelihood of students using the on-campus food pantries identified a significant model (χ^2^(16) = 42.729, *p* ≤ 0.001). The model explained 17% (Nagelkerke R^2^) of the variance in food pantry use and correctly classified 83% of cases. Among all demographic factors, race, education level, and food security status were significant predictors of food pantry use. Specifically, Asian students (OR = 3.415, *p* = 0.014) were 3.41 times more likely to use on-campus food pantries compared to White students. The students in the first year (OR = 0.078, *p* = 0.005), sophomore (OR = 0.134, *p* = 0.012), and junior (OR = 0.266, *p* = 0.050) classes were less likely to use the on-campus food pantries than Ph.D.-level graduate students. Finally, students with low food security (OR = 3.413, *p* = 0.004) were 3.41 times more likely to use the pantries, and students with very low food security (OR = 5.024, *p* < 0.001) were 5.03 times more likely to use the on-campus food pantries compared to students with high food security (Table 4).

Another logistic regression analysis was conducted to determine the associations among demographic factors and food security status on the likelihood of being aware of the on-campus food pantries’ services. The overall logistic regression model was significant (χ^2^(16) = 29.703, *p* = 0.020). The model explained 7.9% (Nagelkerke R^2^) of the variance in food pantry awareness and correctly classified 93%. Among all the predictors, only race and living arrangement were statistically significant. Specifically, Asian students (OR = 2.400, *p* = 0.011) were 2.4 times more likely to be aware of on-campus food pantry services compared to White students. Students who live on-campus (OR = 2.100, *p* = 0.009) were 2.1 times more likely to be aware of on-campus food pantry services compared to students who live off-campus (Table 5).

### 3.3. Barriers to Access the Food Pantries

When students were asked whether they thought there were barriers to accessing the on-campus food pantries, almost one-third answered yes (26.9%; Table 3). Additionally, a multinomial logistic regression was conducted to predict barriers to accessing the pantries from the following demographic factors, age, race, school year, living arrangement, and food security status. The barrier answer “No, I do not think there are barriers” was used as a reference so that the other two levels of outcome measures were compared with the reference. The results showed that the overall model was significant and thus fits the data, χ^2^(26) = 123.145, *p* ≤ 0.001, Nagelkerke R^2^ = 0.268 (see Table 6). The model showed that age, race/ethnicity, and food security status predicted food pantry barriers for “no, I do not need the pantries”. Specifically, a lower proportion of Hispanic/Latino (OR = 0.378, *p* = 0.006) and African American (OR = 0.214, *p* = 0.004) than White students reported that they did not need the food pantries than they did not think barriers existed. In addition, low food secure (OR = 0.046, *p* ≤ 0.001) and very low food secure (OR = 0.227, *p* < 0.001) students were less likely to report they did not need the food panties compared to the high food secure students, suggesting more high food secure students did not need food pantries, whereas more low and very low secure students did not think there were barriers. Moreover, older students were more likely to report that they did not need food pantries (OR = 1.318, *p* = 0.039). When examining students who thought there were barriers to the food pantry versus no barriers, first-year students (OR = 0.241, *p* = 0.041) and junior (OR = 0.361, *p* = 0.041) students were less likely than doctoral students to report there were barriers than no barriers on the food pantry. Moreover, a greater proportion of low food secure (OR = 1.937, *p* = 0.020) students were more likely to report there were barriers compared to high food secure students. Out of those 130 students, 103 students provided feedback on barriers (Table 7). Most of the students reported difficulties with time, transportation, food pantries’ hours of operation, and social stigma. Some of the most frequent nodes were as follows.

“Lack of information about the pantries’ existence, operation, and eligibility” was a commonly reported barrier. Some quotes associated with this barrier were as follows: “Not having enough information, not knowing where to go”, “I am unsure of the hours the pantry is open”, “I thought we could not use the pantries if we did not qualify. Or have some sort of proof that we desperately needed to use the pantry”, “Not being aware of them and, as an international student, I thought that I wasn’t eligible”, “don’t know about it; are there requirements to use it or can anybody get food?”

“Social stigma of being food insecure” was another commonly reported barrier. Some quotes associated with this barrier were as follows: “Embarrassment that other student might think I am poor”, “possibly judgement from other students, and/or having to justify the need which can feel embarrassing”.

“Difficulties with transportation” and “lack of time” were other common barriers. One student said, “after receiving the food, students who don’t have a car have a hard time carrying food back home”. Another student said, “I was not able to make it to the pantries during my time at school cause I had to be at work and they weren’t close or open when I got there in the mornings”.

Finally, some students reported the barrier “feeling like I do not deserve or need it”. A sample quote of a student with this view is, “You need to be a certain amount of poor like I can sometimes afford food but sometimes not but I don’t want to take away from those poorer than me”.

### 3.4. Students’ Suggestions to Improve the On-Campus Food Pantries and Wanted Food Items

Only 35 (7%) students answered the question about suggestions to improve the on-campus food pantry, of which almost half (*n* = 15) did not provide any suggestions (Supplemental Table S1). The respondents suggested that the food pantries should have more flexibility of access, should clear out expired food, increase marketing and advertising, and invest in destigmatizing food insecurity. Some students wrote, “I feel like if there were posters or a way to make food-insecurity less of a burden, I would be more likely to come daily. I come every now and then because sometimes I feel embarrassed. Other than that, I really appreciate both food pantries”, “Letting more people know about it because I don’t think a lot of students know. I told my friend about it because she was also struggling, now she has food”, “Sometimes I only have 15 min of free time that I could use in between classes. It would be helpful if I could place an order online of the food items I wanted to pick and have a time set when I could pick up those items”.

In regard to food items, 42 (8%) provided their opinion (Supplemental Table S2). The most common suggestions were fresh fruits and vegetables, meats, dairy, eggs, fresh food, and even water bottles. In general, the most wanted food items were perishable, fresh food such as milk, yogurt, fruits, vegetables, bread, eggs, raw meats, and even meals. “I wish there were fresh fruit or if there were announcements saying they were giving out fresh food this week and I would go and get them”, “I [no] it’s difficult for food pantries to offer fresh food, especially a campus food pantry, but some produce would be very beneficial in your food pantry”, “Less canned food and more natural products would be wonderful”. Some students also complained about expired food as quoted “Everything it has been offered is good, however the quality sometimes is off, I have gotten rancid nuts 3 times, do not get me wrong it is a relief having this opportunity, however sometimes the products are in bad condition as in “too old” or “convenient brands” which have poor quality and bad products. I recently got tuna cans and the tuna had already gone bad”.

### 3.5. Food Items Provided by the School-Sponsored On-Campus Food Pantry-Inventory

The food pantry inventory was filled out for the school-sponsored food pantry at three time points throughout the Fall semester: beginning, mid-semester, and end-of-semester. Overall, all three inventories showed the pantry did not provide fresh food, such as fruits, vegetables, and eggs; rather, most food items were shelf-stable. Within the fruits and vegetable section, at all time points, the pantry provided canned fruits and vegetables, while dried fruits, frozen carrots, and tomato sauce were available only at mid-semester. The other healthy items listed in the inventory were not available at any point. Among items not listed in the healthy inventory list, the pantry had jelly and apple sauce (Supplemental Table S3).

The healthy inventory grain list emphasized the importance of whole grains, yet the food pantry had few whole grain options available at all time points. Oatmeal and whole-grain pasta were available at the beginning and mid-semester. Instead, the pantry had refined grains such as enriched rice, white spaghetti, yellow corn tortillas, granola bars, pancake mix, breakfast cereals, and non-sugar cereals available. Regarding protein options, the on-campus food pantry had good availability of healthy options at all three time points. It had most of the listed items such as canned beans, canned stews, dried beans and peas, canned chicken and tuna, nuts, and peanut butter. Among the protein items not listed in the inventory, the pantry offered canned pork and beans, chicken soup, tuna casserole, and cheeseburger macaroni.

The pantry did not provide any of the dairy, fat and oils, and spices and condiments item options listed on the healthy inventory. Finally, the pantry offered some other food items such as popped popcorn, lentils, canned meals (spaghetti in marinara sauce, mini ravioli soup, beef lasagna), dried meals (macaroni and cheese, stroganoff pasta in a creamy white sauce), trail mix, original syrup, vegetable soup, chicken broth, chips—corn tortilla, frozen chicken patties, taco shells, butter, and snack mix.

## 4. Discussion

Interventions to alleviate food insecurity among college students have been poorly explored in the literature [8] despite the large body of studies showing their need [1,2,20]. To the best of our knowledge, very few studies have assessed on-campus food pantries as a food security resource for college students [11]. Thus, findings from this study provide needed information about college students’ utilization of on-campus food pantries, including barriers to use and an assessment of the quality of the foods offered by the pantries.

This study highlights a current high prevalence of food insecurity of 49.2% among TWU college students. Very low food security was predicted by race and ethnicity (multiple races). The on-campus food pantries were mostly used by students who identified as Asian, undergraduates, and students who had low and/or very low food security. Asian students and students living on campus were more aware of the on-campus food pantries’ existence. One-third of the participants agreed that there are barriers to accessing the pantries and reported some of them, such as “lack of information about the pantries’ existence, operation, and eligibility”, “social stigma of being food insecure”, “difficulties with transportation”, and others. Most of the participants suggested the food pantries could provide fresh food items and could improve their advertising to ensure increased student outreach. Finally, the sponsored on-campus food pantry inventory showed that most of the food available was shelf-stable items as expected, with a lack of whole grains and fresh produce.

Consistent with past studies on this campus, [21,22] about half of the participants were food insecure (49.2%), although the rate was somewhat higher compared to past surveys (44%) [21]. This rate is similar to other studies reporting mean rates across campuses [1,2]. Moreover, consistent with the other food insecurity literature, race/ethnicity was associated with food insecurity, [3,5,6,13,23] with students identifying themselves as “multiple-race” being more likely to be very low food secure than White students, and undergraduate students (sophomore, junior, and senior) were more likely to be food insecure compared to graduate students. In other studies, the races/ethnicities most correlated with food insecurity were African American [3,5,6,13,21] and Hispanic [6,13]. In this study, most of the “multiple-race” students identified themselves as White + Hispanic, showing agreement with the association between being Hispanic and food insecurity. In addition, the lack of significance between food insecurity and Black students in this study might be due to the low representation of Black students (12.7%) compared to the overall student body (17.1%).

Other studies [13,23,24,25], including one at TWU [21], found that first-year students were at higher risk for food insecurity than other undergraduate students [13,23,24,25]. In fact, past TWU food security assessments showed first-year students at higher risk for food insecurity [21]. In the present study, first-year students did not have higher rates compared to the other students, but other undergraduate students did have higher food insecurity compared to graduate students. Since the institution’s student body changes every semester, and TWU has invested in food security resources for its community (food pantries, mobile food pantries, financial advising, and starting a Swipe Out Hunger student organization), it is possible that food insecurity among first-year students might have decreased from 2019 [21] to 2022. However, consistent with other studies at TWU, undergraduate students remain more likely to be food insecure than graduate students, pointing to the need to give attention to that specific population [13,21,26].

In the past two years since the COVID-19 pandemic started, college students have faced financial hardships that might increase their food insecurity risk and need for food assistance [26,27]. Thus, it is expected that students with food insecurity are willing to take advantage of the free food resources available to them, specifically on-campus resources such as food pantries. This study found that the on-campus food pantries are most used by students with food insecurity, Asian students, and undergraduates (first-year, sophomore, and junior). In addition, Asian students and students living on campus were more likely to be aware of the existence of the on-campus food pantries. Despite the food pantry use and awareness predictors found in this study, the majority of the participants had never used the food pantries (89.8%), and almost half were unaware of the food pantries’ existence (47.8%). Reasons for this lack of use among the TWU students might include higher rates of lack of awareness, perceived barriers to using the pantries, and lack of desired items.

In this study, half of the participants were unaware that TWU Denton has two food pantries, and the majority of participants had never used them. These rates of awareness were similar to a study that assessed first-year students at eight U.S. universities (56%) and lower than in a study at the University of Florida [11] (70%). Similar to the study at the University of Florida, food-insecure students were more likely to use OCFP in this study compared to high food secure students (*p* < 0.001), yet few students reported using the OCFP in this study (10.2% and in the study at the University of Florida (15.6%) [11].

Almost one-third of the participants believed there were barriers to utilizing the pantries, in contrast to the University of Florida study, in which only 12.7% of the participants believed there were barriers to using the pantry. In the University of Florida study, the view of barriers was associated with food security status, with a higher proportion of food-insecure students reporting more barriers to using the pantry compared to food secure students [11], which was similar to the present study, in which low food insecure students were more likely to report barriers to access the pantries compared to high food security students. The barriers cited by TWU students were consistent with the literature, with TWU students also indicating social stigma, inconvenient hours of operation, self-identity [11], food pantry location [13], and lack of information on how to access it [11,13] as potential barriers. Studies with other populations also showed similar barriers to using pantries, such as transportation, advertising, and societal stigma [28].

Another potential reason students do not utilize the pantries might be the availability of food items. In this study, the majority of the students who have used the on-campus pantries reported they were satisfied with the food items, yet they suggested that the food pantry should provide fresh food items, fruits, vegetables, dairy, eggs, and even water bottles. In fact, the TWU food pantries mostly provide non-perishable food items with some exceptions, and specifically, the school-sponsored food pantry that participated in the food pantry inventory showed the pantry lacking many food items students want as well as food items considered healthy according to the inventory list, including limited whole grains, fresh produce, reduced-fat dairy, seasoning, herbs, and other items [17]. The lack of fresh food in the pantries does not only happen at TWU but also occurs in many other pantries [29]. Some of the barriers pantries face to offering fresh food items are the availability of storage, lack of equipment, and difficulty with transportation [29].

Regardless of the lack of some food items, the TWU food pantry, reported in the inventory, is a client-choice pantry, meaning students can choose what they want, which is recognized as a better approach than traditional food pantries. Moreover, it offers a variety of food items throughout the semester and has tried different ways to market the pantry, such as social media, emails, and the TWU website. However, among the suggestions to improve the TWU food pantries, the participants suggested that the pantry should increase the marketing and advertising because they believe many students do not know about the pantry, which is true based on our data on food pantry awareness presented before.

This study has the following strengths. First, it provided updated data on food security status among TWU college students, indicating that food insecurity might change year to year, but undergraduate students remain a higher risk population compared to graduate students at this time. Second, this study contributed to the scarce literature on food pantry utilization among college students, which is an important emergency resource to alleviate acute food insecurity. Third, it had a good sample size of 524 participants. Fourth, this study used a mixed-method approach that not only assessed quantitative methods but also applied thematic analysis to deeper describe students’ opinions on the study’s subject matter. Finally, this study assessed the on-campus food pantry food availability by applying an inventory completed personally by the researcher in order to further explain students’ food needs on the TWU campus.

While this study has strengths, it also has limitations. First, the participants may not represent the total population of TWU students since some demographic populations were overrepresented. For example, full-time students composed 84.7% of this study’s population compared to 52.9% of TWU’s population. However, other groups were underrepresented. For example, students working on master’s degrees composed 17.5% of this study’s population compared to 27.6% of the TWU population. There were also fewer Black students in this sample: 12.9% compared to 17.1% at TWU. Second, few students were aware of the pantries, which was a prerequisite question to answer the open-ended questions about the views of pantry users regarding suggestions and food availability, resulting in a small number of respondents that might not have been fully representative of the TWU student body or of pantry users. The same happened to the core question of this study “what are the barriers to access the pantries?” Since only one-third of the participants agreed that there were barriers to accessing the pantries, the follow-up question about the pantries had few responses. Finally, these findings may not be generalizable to college students across the United States and in other countries as our sample is limited to one primarily female, diverse, a state-funded university located in Texas, U.S.

## 5. Conclusions

In conclusion, this study found that food insecurity remains an urgent problem at TWU since the prevalence has remained high since 2019 despite the institutions’ efforts to reduce it. This study shows that one of those resources has not been utilized as expected, which might impede improvements in food security among students. Students pointed out that there are barriers to access the on-campus food pantries that might increase this lack of use and constant high prevalence of food insecurity status, such as difficulties with time, transportation, food pantries’ hours of operation, and social stigma. Those barriers were also found in other studies, meaning that they must be addressed for students to be able to utilize this resource.

Therefore, we recommend that on-campus food pantries should invest in some additional resources to improve their outreach. First, they should invest in substantially increased advertisement to ensure students know about the pantries. The advertisement could include repeated biweekly emails with the food pantry information of location, hours of operation, how to access it, eligibility, and food items available, and participation in tabling events, such as students’ fairs, block parties, students’ orientation, and others that each institution might have. Second, hours of operation could expand and include weekend access. Third, students complained about difficulties with transportation. Perhaps, the pantries could partner with the Denton County Transportation Authority (DCTA) on transportation to help students with this matter. Another solution could be to start a friend ride campaign for students who have a car to help students who do not. Fourth, the pantries could work together with Counseling and Psychological Services to normalize accessing food assistance, re-branding the pantry as a food grocery, and switching to client-choice pantries, thus reducing social stigma. Fifth, the on-campus food pantries should take into consideration students’ food needs and preferences to improve de availability of food options students suggested in this study, such as fresh food and fresh produce. In order to have those items available, food pantries could partner with local farmers/farmers’ markets, community gardens, local food banks, or even start a community garden on campus where students could volunteer.

Finally, while the accessed pantry in this study did not provide some of the healthy food items listed in the inventory, it did provide a variety of food items that could be easily incorporated into a healthy diet, such as canned fruits and vegetables, and canned meats, yet students might face personal barriers in utilizing those food items due to lack of cooking skills and nutrition knowledge. Thus, we recommend that the pantries could have nutrition students as volunteers to provide in-person nutrition education and food literacy in general, including budgeting and encouraging students to take advantage of institutional resources to help them learn how to budget and have a healthier diet, such as participating in cooking classes, nutrition-related events, and looking for professional financial advising from the financial advising department.

## Figures and Tables

**Table 1:** Food Security Status and Demographic Characteristics of Survey Participants versus TWU Denton Students Body’s Characteristics.

Variables	*n*	%	TWU Student Body in Fall 2021 (%)
**Food Security Status**			
High Food Security	269	50.9	
Low Food Security	130	24.6	
Very Low Food Security	130	24.6	
**Gender**			
Male	31	6.1	11.4
Female	477	93.9	88.6
**Age**			
18–19	122	23.2	15.6
20–21	114	21.7	15.5
22–25	127	24.2	23.0
26–29	68	13.0	11.8
30 or older	94	17.9	24.7
**Race**			
White	212	40.1	39.5
African American	68	12.9	17.1
Hispanic/Latino	119	22.5	30.1
Asian	58	11.0	7.1
Multiple race/ethnicity	55	10.4	5.8
**School Enrollment**			
Full-time	448	84.7	52.9
Part-time	81	15.3	47.1
**School Year**			
First-year	87	16.5	19.5
Sophomore	77	14.6	10.7
Junior	103	19.6	16.1
Senior	120	22.8	20.5
Graduate (Master’s degree)	92	17.5	27.6
Graduate (Doctoral degree)	47	8.9	4.4
**Living arrangement**			
On-campus	145	27.6	11
Off-campus	381	72.4	88
**Work Hours per week**			
None	133	25.6	
Part-time (0–20 h per week)	210	40.5	
Full-time (21+ hours per week)	176	33.9	
**International student**			
Yes	15	2.8	2.0
No	513	97.2	
**Having children under 18y in the household**			
Yes	97	18.4	
No	429	81.6	
**Single parent**			
Yes	31	5.9	
No	496	93.8	

**Table 2:** Summary of Multinomial Regression Predicting Food Security According to Demographic and Other Factors.

Variables	β	SE	Wald	OR	95% CI of OR		*p*
					Lower	Upper	
**Low Food Security ^a^**							
Age	0.010	0.125	0.007	1.010	0.792	1.290	0.934
Multiple races ^b^	0.093	0.410	0.051	1.097	0.491	2.450	0.821
Asian ^b^	−0.758	0.437	3.014	0.469	0.199	1.103	0.083
Hispanic/Latino ^b^	0.165	0.281	0.344	1.179	0.679	2.048	0.557
African American ^b^	0.219	0.372	0.347	1.245	0.600	2.584	0.556
First-Year ^c^	−0.023	0.617	0.001	0.977	0.291	3.277	0.970
Sophomore ^c^	0.593	0.539	1.214	1.810	0.630	5.202	0.271
Junior ^c^	0.760	0.483	2.471	2.138	0.829	5.516	0.116
Senior ^c^	0.112	0.471	0.056	1.118	0.445	2.813	0.812
Graduate (Master’s) ^c^	0.047	0.469	0.010	1.048	0.419	2.626	0.920
Living on-campus ^d^	0.437	0.324	1.824	1.548	0.821	2.921	0.177
Working hours (None) ^e^	−0.142	0.323	0.193	0.868	0.461	1.634	0.660
Working hours (part-time) ^e^	−0.197	0.283	0.485	0.821	0.472	1.429	0.486
Having Children under 18y living in the household (Yes) ^f^	−0.122	0.318	0.147	0.885	0.475	1.651	0.702
**Very Low Food Security ^a^**							
Age	0.219	0.126	3.030	1.245	0.973	1.594	0.082
**Multiple races ^b^**	0.797	0.367	4.707	**2.218**	1.080	4.556	**0.030**
Asian ^b^	−0.597	0.425	1.970	0.551	0.239	1.267	0.160
Hispanic/Latino ^b^	−0.424	0.332	1.624	0.655	0.341	1.256	0.202
African American ^b^	0.685	0.356	3.706	1.985	0.988	3.988	0.054
First-Year ^c^	1.305	0.719	3.288	3.686	0.900	15.099	0.070
**Sophomore ^c^**	1.602	0.644	6.191	**4.962**	1.405	17.525	**0.013**
**Junior ^c^**	1.651	0.595	7.698	**5.214**	1.624	16.744	**0.006**
**Senior ^c^**	1.612	0.563	8.210	**5.013**	1.664	15.099	**0.004**
Graduate (Master’s) ^c^	0.728	0.577	1.594	2.071	0.669	6.413	0.207
Living on-campus ^d^	0.435	0.346	1.586	1.546	0.785	3.043	0.208
Working hours (None) ^e^	−0.496	0.336	2.174	0.609	0.315	1.177	0.140
Working hours (part-time) ^e^	−0.386	0.285	1.842	0.680	0.389	1.187	0.175
Having Children under 18y living in the household (Yes) ^f^	−0.402	0.338	1.411	0.669	0.345	1.299	0.235

Note. χ^2^(28) = 50.06, *p* = 0.005, Nagelkerke R^2^ = 0.112 ^a^ Compared to High Food Security. ^b^ Compared to White. ^c^ Compared to Graduate (Ph.D. degree). ^d^ Compared to Living off-campus. ^e^ Compared to Working hours (full-time). ^f^ Having Children under 18y living in the household (No).

**Table 3:** Description of Students’ On-Campus Food Pantries Awareness, Use of, Satisfaction with, and Perceived Barriers to Use.

Variables	*n*	%
**Food pantry awareness**		
Yes	271	52.2
No	248	47.8
**Food pantry use**		
Yes. I use or used food from the Minerva’s Market pantry to supplement regular food needs	18	3.5
Yes. I use or used food from the Social Work Food Pantry to supplement regular food needs	11	2.2
Yes. I use or used food from both TWU food pantries to supplement regular food needs	15	2.9
Yes. I use or used food from the Minerva’s Market pantry as the sole source of food	1	0.2
Yes. I use or used food from the Social Work Food Pantry as the sole source of food	1	0.2
Yes. I use or used food from both TWU food pantries as the sole source of food	6	1.2
No. I have not	458	89.8
**Students’ satisfaction**		
Yes. I was satisfied with the Food items both TWU Food pantries offer	23	47.9
Yes. I was satisfied only with the food items the Minerva’s Market offers	14	29.2
Yes. I was satisfied only with the food items the Social Work Food Pantry offers	8	16.7
No. I was not satisfied	3	6.3
**Barriers to accessing the on-campus food pantries**		
No, I do not need the food pantries	114	23.6
No, I do not think there are barriers	240	49.6
Yes. There are barriers	130	26.9

**Table 4:** Summary of Logistic Regression Predicting Food Pantry Use by Demographic Factors and Food Security Status.

Variables	β	Wald	OR	95% CI		*p*
				Lower	Upper	
Age	−0.264	1.737	0.768	0.518	1.137	0.187
Multiple races ^a^	0.487	0.861	1.628	0.582	4.558	0.353
**Asian ^a^**	1.228	6.007	**3.415**	1.279	9.118	**0.014**
Hispanic/Latino ^a^	0.661	2.359	1.936	0.833	4.498	0.125
African American ^a^	0.036	0.004	1.037	0.354	3.042	0.947
**First-year ^b^**	−2.553	7.744	**0.078**	0.013	0.470	**0.005**
**Sophomore ^b^**	−2.010	6.269	**0.134**	0.028	0.646	**0.012**
**Junior ^b^**	−1.324	3.846	**0.266**	0.071	0.999	**0.050**
Senior ^b^	−0.617	1.072	0.540	0.168	1.735	0.301
Master’s degree ^b^	−1.068	2.503	0.344	0.092	1.290	0.114
Living on-campus ^c^	0.632	2.178	1.882	0.813	4.358	0.140
Working hours (none) ^d^	0.196	0.162	1.216	0.469	3.153	0.687
Working hours (part-time) ^d^	0.250	0.397	1.285	0.589	2.801	0.529
Having children under 18y living in the household (yes) ^e^	−0.666	1.339	0.514	0.166	1.587	0.247
**Food security status (low food security) ^f^**	1.228	8.417	**3.413**	1.489	7.821	**0.004**
**Food security status (very low food security) ^f^**	1.614	15.042	**5.024**	2.222	11.357	**<0.001**

Note.: χ^2^(16) = 42.729, *p* ≤ 0.001, Nagelkerke *R*^2^ = 0.175. ^a^ Compared to White. ^b^ Compared to graduate (Ph.D. degree). ^c^ Compared to living off-campus. ^d^ Compared to working hours (full-time). ^e^ Compared to having children under 18y living in the household (No). ^f^ Compared to high food security.

**Table 5:** Summary of Logistic Regression Predicting Food Pantry Awareness by Demographic Factors and Food Security Status.

Variables	B	Wald	Odd Ratio	95% CI		*p*
				Lower	Upper	
Age	0.033	0.105	1.034	0.846	1.263	0.746
Multiple races ^a^	0.111	0.114	1.117	0.587	2.128	0.736
**Asian ^a^**	0.875	6.420	**2.400**	1.219	4.724	**0.011**
Hispanic/Latino ^a^	−0.119	0.231	0.887	0.545	1.445	0.631
African American ^a^	−0.060	0.040	0.941	0.520	1.705	0.842
First-year ^b^	0.009	0.000	1.009	0.363	2.807	0.986
Sophomore ^b^	−0.405	0.793	0.667	0.273	1.627	0.373
Junior ^b^	−0.276	0.457	0.758	0.340	1.691	0.499
Senior ^b^	0.224	0.337	1.251	0.587	2.665	0.561
Master’s degree ^b^	−0.386	0.993	0.679	0.318	1.453	0.319
**Living on-campus ^c^**	0.742	6.879	**2.100**	1.206	3.656	**0.009**
Working hours (none) ^d^	−0.174	0.408	0.840	0.493	1.433	0.523
Working hours (part-time) ^d^	0.228	0.954	1.257	0.795	1.987	0.329
Having children under 18y living in the household (yes) ^e^	−0.110	0.171	0.896	0.533	1.507	0.679
Food security status (low food security) ^f^	−0.066	0.080	0.936	0.595	1.475	0.777
Food security status (very low food security) ^f^	−0.078	0.103	0.925	0.576	1.486	0.748

Note.: χ^2^(16) = 29.703, *p* = 0.020, Nagelkerke *R*^2^ = 0.079. ^a^ Compared to White. ^b^ Compared to graduate (Ph.D. degree). ^c^ Compared to living off-campus. ^d^ Compared to working hours (full-time). ^e^ Compared to having children under 18y living in the household (No). ^f^ Compared to high food security.

**Table 6:** Summary of Logistic Regression Predicting Food Pantry Barriers by Demographic Factors and Food Security Status.

Variables	B	Wald	Odd Ratio	95% CI		*p*
				Lower	Upper	
**No, I do not need the food pantries**						
**Age**	0.276	4.268	1.318	1.014	1.713	**0.039**
Multiple races ^a^	0.181	0.170	1.198	0.508	2.826	0.680
Asian ^a^	−0.507	1.583	0.602	0.273	1.327	0.208
**Hispanic/Latino ^a^**	−0.973	7.574	**0.378**	0.189	0.756	**0.006**
**African American ^a^**	−1.544	8.263	**0.214**	0.075	0.612	**0.004**
First-year ^b^	−0.440	0.406	0.644	0.167	2.490	0.524
Sophomore ^b^	−0.055	0.008	0.947	0.279	3.208	0.930
Junior ^b^	−0.270	0.252	0.763	0.266	2.192	0.616
Senior ^b^	−0.473	0.868	0.623	0.230	1.686	0.351
Master’s degree ^b^	−0.224	0.201	0.799	0.300	2.131	0.654
Living on-campus ^c^	0.289	0.522	1.335	0.610	2.920	0.470
**Food security status (low food security) ^d^**	−3.082	24.158	**0.046**	0.013	0.157	**<0.001**
**Food security status (very low food security) ^d^**	−1.485	19.732	**0.227**	0.118	0.436	**<0.001**
**Yes, there are barriers**						
Age	0.172	2.013	1.188	0.937	1.506	0.156
Multiple races ^a^	−0.301	0.498	0.740	0.321	1.708	0.480
Asian^a^	0.191	0.240	1.210	0.564	2.597	0.624
Hispanic/Latino ^a^	0.027	0.008	1.027	0.564	1.870	0.931
African American ^a^	−0.174	0.232	0.840	0.415	1.704	0.630
**First-year ^b^**	−1.268	4.180	**0.281**	0.083	0.949	**0.041**
Sophomore ^b^	−1.053	3.535	0.349	0.116	1.046	0.060
**Junior ^b^**	−1.020	4.165	**0.361**	0.135	0.960	**0.041**
Senior ^b^	−0.492	1.138	0.611	0.247	1.510	0.286
Master’s degree ^b^	−0.913	3.660	0.401	0.158	1.023	0.056
Living on-campus ^c^	0.381	1.261	1.464	0.753	2.850	0.261
**Food security status (low food security) ^d^**	0.661	5.387	**1.937**	1.108	3.386	**0.020**
Food security status (very low food security) ^d^	0.029	0.010	1.030	0.578	1.834	0.921

Note.: χ^2^(26) = 123.145, *p* ≤ 0.001, Nagelkerke *R*^2^ = 0.268. ^a^ Compared to White. ^b^ Compared to graduate (Ph.D. degree). ^c^ Compared to living off-campus. ^d^ Compared to high food security. The entire model is compared to “No, I do not think there are barriers”.

**Table 7:** Thematic Analysis of Barriers to Accessing the Food Pantries Extracted from Open-Ended Question Responses.

Themes	Sub-Themes
Barriers to accessing the food pantries	Being a full-time student
	Difficulties with transportation
	Embarrassing questions to access the pantry
	Feeling like I do not deserve or need it
	Lack of info about the pantry’s existence, operation, and eligibility
	Lack of time
	Food pantry location
	Poor food quality
	Reduced hours of operation
	Social stigma of being food insecure

## Data Availability

Not applicable.

## Supplementary Materials

The following supporting information can be downloaded at: https://www.mdpi.com/article/10.3390/nu14142807/s1, Table S1: Thematic Analysis of Students’ Suggestions to Improve On-Campus Food Pantries from Open-Ended Questions Responses; Table S2: Thematic Analysis of Students’ Suggestion of Food Items They Would Like the On-Campus Food Pantries to Offer from Open-Ended Questions Responses; Table S3: Description of Food Availability in One On-Campus Food Pantry during Fall 2021.
