# Supplementary material for: College Campus Food Pantry Program Evaluation: What Barriers Do Students Face to Access On-Campus Food Pantries?
Source: Nutrients. 2022 Jul 8;14(14):2807. doi: 10.3390/nu14142807 (PMC9324299; doi:10.3390/nu14142807)
Supplement: Supplementary file 1 [file nutrients-14-02807-s001.zip › Supplemental Table S1.pdf]

Supplemental Table S1: Thematic Analysis of Students' Suggestions to Improve On-Campus Food Pantries from Open-Ended Questions Responses.

**Themes**	**Sub-themes**
Suggestions to improve the on-campus food pantries	Food for kids
	Investing in destigmatizing food insecurity
	Marketing the food pantries more often
	More flexibility of access
	No suggestions
	Non-perishable items
	To clear out expired food
	Winter clothing
	Feminine hygiene products
