# Supplementary material for: College Campus Food Pantry Program Evaluation: What Barriers Do Students Face to Access On-Campus Food Pantries?
Source: Nutrients. 2022 Jul 8;14(14):2807. doi: 10.3390/nu14142807 (PMC9324299; doi:10.3390/nu14142807)
Supplement: Supplementary file 1 [file nutrients-14-02807-s001.zip › Supplemental Table S2.pdf]

Supplemental Table S2: Thematic Analysis of Students' Suggestion of Food Items They Would Like the On-Campus Food Pantries to Offer from Open-Ended Questions Responses.

Theme	Sub-themes	Codes
Suggestion of food items	Dairy	
	Drinks	
	Fruits and Vegetables	Fresh Fruits or fruits
		Fresh vegetables or vegetables
		Potatoes
	Grains	Beans
		Bread
		Cereals
		Granola
		Pasta or instant noodles
		Rice
	Nuts	
	Other food items	Add water mixtures
		Fresh food
		Meals
		More canned food options
		More healthier options
		Non-expired food
		Seasonings and soup stock
	Proteins	Eggs
		Ham
		Meats
		Protein shakes
	Snacks	
