# Supplementary material for: College Campus Food Pantry Program Evaluation: What Barriers Do Students Face to Access On-Campus Food Pantries?
Source: Nutrients. 2022 Jul 8;14(14):2807. doi: 10.3390/nu14142807 (PMC9324299; doi:10.3390/nu14142807)
Supplement: Supplementary file 1 [file nutrients-14-02807-s001.zip › Supplemental Table S3.pdf]

Supplemental Table S3: Description of Food Availability in One On-Campus Food Pantry during Fall 2021.

**Food Items**	**Beginning**<br>**Yes/No**	**Mid**<br>**Yes/No**	**End**<br>**Yes/No**
**Fruits and Vegetables**			
Canned fruits and vegetables	Yes	Yes	Yes
Diced tomatoes	No	No	No
Dried fruits	No	Yes	No
Fresh fruits or vegetables	No	No	No
Frozen fruits or vegetables	No	Yes	No
Fruit juice (100%)	No	No	No
Shelf-stable fruit cups	No	No	No
Spaghetti sauce	No	No	No
Tomato sauce	No	Yes	No
Vegetable juice (100%)	No	No	No
**Grains**			
Brown rice or barley	No	No	No
Low-sugar, high-fiber cereal	No	No	Yes (non-sugar)
Oatmeal	Yes	Yes	No
Quinoa	No	No	No
Whole grain breads	No	No	No
Whole grain crackers	No	No	No
Whole grain pasta	Yes	Yes	No
Whole grain tortillas	No	No	No
Whole wheat flour	No	No	No
**Protein**			
Canned beans	Yes	Yes	Yes
Canned chilis and stews	No	Yes	Yes
Dried beans and peas	Yes	Yes	Yes
Canned chicken, tuna, or salmon	Yes	Yes	Yes

**Food Items**	**Beginning Yes/No**	**Mid Yes/No**	**End Yes/No**
Eggs	No	No	No
Nuts	No	Yes	No
Peanut butter or other nut butter	Yes	Yes	Yes
**Dairy**			
Boxed shelf stable milk	No	No	No
Calcium-fortified milk alternatives such as soy, rice, or almond milks	No	No	No
Dry milk	No	No	No
Evaporated milk	No	No	No
Low-fat cheese	No	No	No
Low-fat plain yogurt	No	No	No
**Fat and Oils**			
Canola oil	No	No	No
Non-stick cooking spray	No	No	No
Olive oil	No	No	No
Vegetable oil	No	No	No
**Spices and condiments**			
Basil	No	No	No
Bay leaves	No	No	No
Black pepper	No	No	No
Cayenne pepper	No	No	No
Chili powder	No	No	No
Cinnamon	No	No	No
Cumin	No	No	No
Curry powder	No	No	No
Dill	No	No	No
Garlic powder	No	No	No
Mustard	No	No	No

Food Items	Beginning<br>Yes/No	Mid<br>Yes/No	End<br>Yes/No
Onion powder	No	No	No
Oregano	No	No	No
Parsley	No	No	No
Red pepper flakes	No	No	No
Rosemary	No	No	No
Salsa (jarred)	No	No	No
Thyme	No	No	No
Vanilla extract	No	No	No
